# VEGF-B targeting by aryl hydrocarbon receptor mediates the migration and invasion of choriocarcinoma stem-like cells

**DOI:** 10.1186/s12935-022-02641-8

**Published:** 2022-06-30

**Authors:** Qianxia Tan, Jingting Cai, Jingping Peng, Cui Hu, ChenChun Wu, Huining Liu

**Affiliations:** Department of Gynecology and Obstetrics, Xiangya Hospital Central South University, 87 Xiangya Road, Kaifu, Changsha, Hunan 410000 People’s Republic of China

**Keywords:** Choriocarcinoma, VEGF-B, Migration, Invasion, Cancer stem cell

## Abstract

Unlike other members of the VEGF family, the function of VEGF-B in tumor progression remains to be elucidated. Thus, the present study aimed to determine the function of VEGF-B in human choriocarcinoma cells by investigating its detailed effects and molecular mechanisms. VEGF-B and aryl hydrocarbon receptor (AhR) expression were evaluated by reverse transcription-quantitative PCR analysis and western blot analysis in JEG-3 cells and choriocarcinoma stem-like cells (CSLCs) and their proliferation, migration, and invasion after the transfection of short hairpin RNA VEGF-B, 2,3,7,8-tetrachlorodibenzo-*p*-dioxin (TCDD; AhR agonist) treatment or StemRegenin 1 (SR1; AhR antagonist) treatment were examined by cell proliferation assay, wound healing assay and Transwell assay. In addition, luciferase reporter analysis and bioinformatics data mining were used to investigate the association between VEGF-B and AhR. Upregulation of VEGF-B and AhR expression was observed in CSLCs. Following VEGF-B knockdown or SR1 treatment, the proliferative, migratory, and invasive abilities of CSLCs were significantly decreased, contrary to the findings after TCDD treatment. It was also found that AhR enhanced VEGF-B transcriptional activity by binding to the relative promoter region. These observations indicated that VEGF-B may be an oncogene that promotes choriocarcinoma cell migration and invasion targeted by AhR. Therefore, targeting VEGF-B may provide a novel therapeutic opportunity for choriocarcinoma.

**Supplementary Information:**

The online version contains supplementary material available at 10.1186/s12935-022-02641-8.

## Background

Choriocarcinoma, primarily located in the uterus, is an aggressive tumor type that belongs to the family of gestational trophoblastic diseases (GTDs) [1, 2]. Choriocarcinoma can spread rapidly and has a ~ 100% mortality rate if metastasis occurs and a ~ 60% rate even if a hysterectomy has been performed for choriocarcinoma without apparent metastasis [3]. The main cause of choriocarcinoma treatment failure is succumbing due to extensive metastasis or the development of drug resistance [4]. Therefore, the metastatic mechanism of choriocarcinoma should be fully investigated and a new approach for reversing migration and invasion should be considered for patients with metastatic choriocarcinoma.

Cancer stem cells (CSCs) include a small number of tumor cell subsets that exhibit stemness [5]. Self-renewal ability and undirected differentiation potential are two characteristics of CSCs, which serve an essential role in promoting tumor progression, antitumor treatment, and tumor metastasis [6–8]. CSCs have been observed in numerous cancer types, such as leukemia [9], breast cancer [10], and colorectal cancer [11]. Previous studies have shown that the VEGF/neuropilin signaling pathway serves a vital role in the initiation of tumor invasion and metastasis involving CSCs, which is independent of promoting angiogenesis and increasing vascular permeability [12, 13]. The hypothesis that tumor cells are fueled by CSCs has led to the concept that anti-CSC therapy could be considered as a possible therapeutic strategy [5]. However, the mechanism of CSCs in the migration and invasion processes of choriocarcinoma remains to be elucidated.

VEGF-B is a member of the VEGF family, which was first isolated from human fibrosarcoma, erythroleukemia cells, and multiple endocrine neoplasia type 1 in 1996 [14]. VEGF-B binds to VEGF-receptor 1 (VEGFR-1) and neuropilin-1 (NRP1) [15]. It is considered that tumor cells release VEGF and placental growth factor prior to invasion and metastasis to promote tumor metastasis to the target organs [12, 16]. VEGF-B participates in the development and progression of numerous cancer types, including breast [17], pancreatic [18], and colorectal [19] cancer. A prospective clinical study showed that higher VEGF-B expression in muscle-invasive bladder cancer is associated with decreased overall survival rate and distant metastasis [20]. Based on these studies, it can be suggested that VEGF-B may act as an oncogene in these cancers. However, its function and the possible underlying mechanisms in choriocarcinoma remain unknown, to the best of the authors’ knowledge.

Aryl hydrocarbon receptor (AhR), a member of the basic helix-loop-helix transcription factor family, is best known for mediating the toxicity and tumor-promoting properties of the carcinogen 2,3,7,8-tetrachlorodibenzo-*p*-dioxin (TCDD). AhR serves a critical role in tumorigenesis-initiation, promotion, progression, and metastasis [21]. It has been shown that treatment of head and neck squamous cell carcinoma with the antagonists of AhR decreased the migration and invasion of these tumor cells [22]. However, the mechanism of AhR in choriocarcinoma metastasis is yet to be fully elucidated.

The present study isolated choriocarcinoma stem-like cells (CSLCs) from JEG-3 cells as previously described [23]. The current study aimed to examine the expression of VEGF-B in JEG-3 cells and CSLCs to determine whether VEGF-B may serve as an oncogene for choriocarcinoma. In addition, the relationship between VEGF-B and the proliferation, migration, and invasion of CSLCs was investigated. By using the UCSC genome browser, it was found that there were AhR-binding sites in the promoter region of VEGF-B and a novel hypothesis was proposed that AhR binding to VEGF-B promoter may mediate the migration and invasion of CSLCs.

## Materials and methods

### Cell culture

The human choriocarcinoma cell line, JEG-3 and the 293 T cell line were obtained from the American Type Culture Collection and maintained in high glucose-DMEM supplemented with 10% FBS (both Gibco; Thermo Fisher Scientific, Inc.), 1% penicillin–streptomycin in a humidified atmosphere containing 5% CO_2_ at 37 ˚C. CSLCs were cultured in a serum-free medium as previously described [23, 24].

TCDD (AccuStandard) and StemRegenin-1 (SR1; Selleck) were stocked and freshly prepared prior to each experiment using DMSO; the DMSO concentration was ~ 0.01%. Live cells were treated with control media (0.01% DMSO), 50 nM TCDD, 100 nM TCDD, 0.75 μM SR1, and 1 μM SR1 for 24 h at room temperature in the following experiments.

### Cell proliferation assay

Cell proliferation was assessed by using the Cell Counting Kit (CCK)-8 assay (Dojindo Laboratories, Inc.) every 24 h, according to the manufacturer’s protocol. Briefly, a total of 2 × 10^3^ cells were seeded in 96-well plates. After being cultured under different experimental conditions, each well was incubated with 10 μl CCK-8 for 2 h at 37˚C. The optical density (OD) of each well was measured at 450 nm using a microplate reader (BioTek Instruments, Inc.).

### Wound healing assay

JEG-3 cells (4 × 10^5^) and CSLCs (2 × 10^5^) were seeded in a 6‐well plate with 10% FBS until the cell monolayer fused > 90%. The cell monolayers were wounded by dragging a 10-μl sterile pipette tip across the middle. Serum-free medium was used to wash the cellular debris and then the cells were cultured in 1% FBS [25] with or without TCDD (50 and 100 nM) and SR1 (0.75 and 1 μM). Following growth for 24 h, cells were observed and imaged under an optical microscope (Leica Microsystems GmbH; magnification, × 100). The migration rate of cells was calculated as follows: Migration rate (%) = (A_0_ − A_n_)/A_0_ × 100%, where A_0_ represents the initial wound width and A_n_ represents the remaining width of the wound at the metering point.

### Transwell invasion assay

Matrigel (BD Biosciences) was obtained to cover the bottom membrane of the 8 μm Millicell System (Millicell; Corning, Inc.). Each Transwell membrane was pre-coated with Matrigel and medium at 37˚C at the ratio of 1:2 at 50 µl. The upper chamber was filled with 2 × 10^4^ cells/ml in serum-free culture media and the lower chamber was filled with DMEM‐high glucose containing 10% FBS. After incubation at 37˚C for 24 h, the cells in the upper chamber were removed with a cotton swab and washed with PBS three times [26]. Then, chambers were stabilized with 4˚C methanol for 20 min and 0.4% trypan blue solution was added for 10 min at room temperature before washing. The stained cells were imaged and counted under an optical microscope (magnification, × 100) in five randomly selected fields.

### RNA extraction and reverse transcription-quantitative (RT-q) PCR

Total RNA was extracted from 1 × 10^7^ cultured cells using 1 ml TRIzol® reagent (Invitrogen; Thermo Fisher Scientific, Inc.) [27]. cDNA was reversely transcribed using a Transcriptor First Strand cDNA Synthesis kit (GeneCopoeia, Inc.). The reaction conditions were as follows: 37˚C for 15 min, 85˚C for 5 s, and 4˚C for termination. qPCR was performed using SYBR PremixEx Taq II (TaKaRa, Inc.) with the ABI 7500 real-time PCR system (Applied Biosystems; Thermo Fisher Scientific, Inc.). The reaction conditions included: 1 cycle of pre-denaturation at 95˚C for 3 min, followed by 45 cycles of 95˚C for 30 s, 60˚C for 30 s and 72˚C for 5 min. RNA extraction, cDNA synthesis, and qPCR were performed according to the manufacturer’s protocols. The relative standard curve method 2^−∆∆Cq^ [28] was used to calculate the relative mRNA expression level, using GAPDH as the reference. The primers obtained from Sangon Biotech Co., Ltd. are shown in Table 1. The assay was performed in triplicate and repeated 3 times.

**Table 1:** The sequences of gene primers

Gene	Primer	Sequence (5'-3')
AhR	Forward	ACGTCAGCAAGTTCACATGG
	Reverse	GTGGCAGCACCCTTTCTATC
VEGF-B	Forward	GAAGACCCAAACCTCTGCAT
	Reverse	GCCTGGACAGTGACAAACAG
GAPDH	Forward	GCTGGCGCTGAGTACGTCGT
	Reverse	TGGGTGTCGCTGTTGAAGTC

*AhR* aryl hydrocarbon receptor

### Western blot analysis

Total proteins were extracted using RIPA lysis buffer (Beyotime Institute of Biotechnology) and quantified using the standard BCA method [29]. Equivalent amounts of protein from each sample (20–50 µg) were loaded onto a 10% SDS-PAGE gel and transferred onto PVDF membranes (Merck KGaA). TBS-Tween-20 (0.1%) supplemented with 5% milk was used for blocking the membrane for 1 h at room temperature and then incubated with the following primary antibodies (all used at a 1:1,000 dilution) at 4˚C overnight: AhR [cat. no. 83200; Cell Signaling Technology, Inc. (CST)], VEGF-B (cat. no. ab110649; Abcam), E-cadherin (cat. no. ab181296; Abcam), vimentin (cat. no. ab217673; Abcam) and GAPDH (cat. no. 5174; CST). Anti-rabbit (cat. no. 7074S; CST) and anti-mouse (cat. no. 7076S; CST) constituted the secondary antibodies at 1:5,000 dilution and were added for 1 h at room temperature, following which bands were detected with an ECL reagent (MilliporeSigma) and exposed using a chemiluminescence system (Syngene Europe). The gray value of each band was measured using ImageJ 1.8.0 software (National Institutes of Health).

### Plasmids

VEGF-B short hairpin RNAs (shRNA/sh) were synthesized by Vigene Biosciences. AhR small interfering RNA (siRNA/si) and the overexpression AhR (OE-AhR) plasmid (NM_001621.5) were provided by Guangzhou RiboBio Co., Ltd. The sequence of VEGF-B shRNA and AhR siRNA are shown in Additional file 1: Table S1. In order to clone the VEGF-B (NM_003377) promoter region, gene-specific primers were designed to amplify a 2.0-kb (-2,000- + 50) genomic region upstream of the VEGF-B gene. For the generation of the luciferase reporter construct, the 2.0-kb VEGF-B promoter fragment was ligated into the pGL3-basic vector (RiboBio Co., Ltd). This plasmid was named VEGFB-wild-type (WT)1. A vector including a 1.0-kb (− 1000 to  + 50) genomic region upstream of the VEGF-B gene was named VEGFB-WT2. The mutant type (VEGF-B Mut) was also cloned into the pGL3-basic vector.

### Transfection

Cells were seeded in 24-well plates at a density of 1 × 10^4^ cells/well and transfected with appropriately concentrated lentivirus VEGF-B shRNAs for gene silencing and non-specific control shRNA (sh-NC) at a multiplicity of infection of 100 in the presence of polybrene (2.5 μg/ml) at 37 °C overnight. Culture medium containing virus was collected at 48 h following transfection, and then maintained at -80 °C until further use. The stably transfected cells were selected with puromycin (1 μg/ml) after 48 h. Lentiviruses were synthesized and cloned into the GV248 vector by Shanghai GeneChem Co., Ltd.

JEG-3 cells (2 × 10^5^ cells/well) were plated in 6-well plates, following which AhR siRNA and the OE-AhR plasmid were transfected into cells at a final concentration of 100 nM, using the Lipofectamine® 3000 reagent (Invitrogen; Thermo Fisher Scientific, Inc.) following the manufacturer’s protocol. Essential experiments were conducted 48 h at 37˚C after transfection.

### Luciferase reporter assay

The luciferase reporter assay (Promega Corporation) was conducted according to the manufacturer’s protocol. A total of 1 × 10^4^ JEG-3 or 293 T cells were seeded per well in 96-well plates, co-transfected with the aforementioned reporter plasmid, siAhR, or OE-AhR, and treated with 100 nM TCDD or 1 μM SR1 at room temperature. After 48 h, cells were lysed and firefly luciferase activity was measured using a Dual Luciferase Reporter Assay system (Promega Corporation) and normalized to *Renilla*.

*Bioinformatics data mining.* Gene Expression Omnibus (GEO) raw data of different cells were downloaded from GEO datasets (http://www.ncbi.nlm.nih.gov/geo/) for use as the samples for correlation analysis. The molecular functional network map of canonical pathways, including physical interactions, co-expression, co-localization, pathway, genetic interactions, shared protein domains, and predicted networks of AhR and VEGF-B, were analyzed using GeneMANIA version 3.5.2 (http://genemania.org/).

### Statistical analysis

The SPSS software package 25.0 (IBM Corp.) was used for statistical analysis. Continuous data were compared using an unpaired Student’s t-test or one-way ANOVA followed by a post-hoc Bonferroni test. The co-expression correlation of AhR and VEGF-B was tested via Spearman correlation analysis. The data are presented as the mean ± SD. P < 0.05 was considered to indicate a statistically significant difference.

## Results

### Proliferation, migration, and invasion of JEG-3 cells and CSLCs

**Fig. 1:**
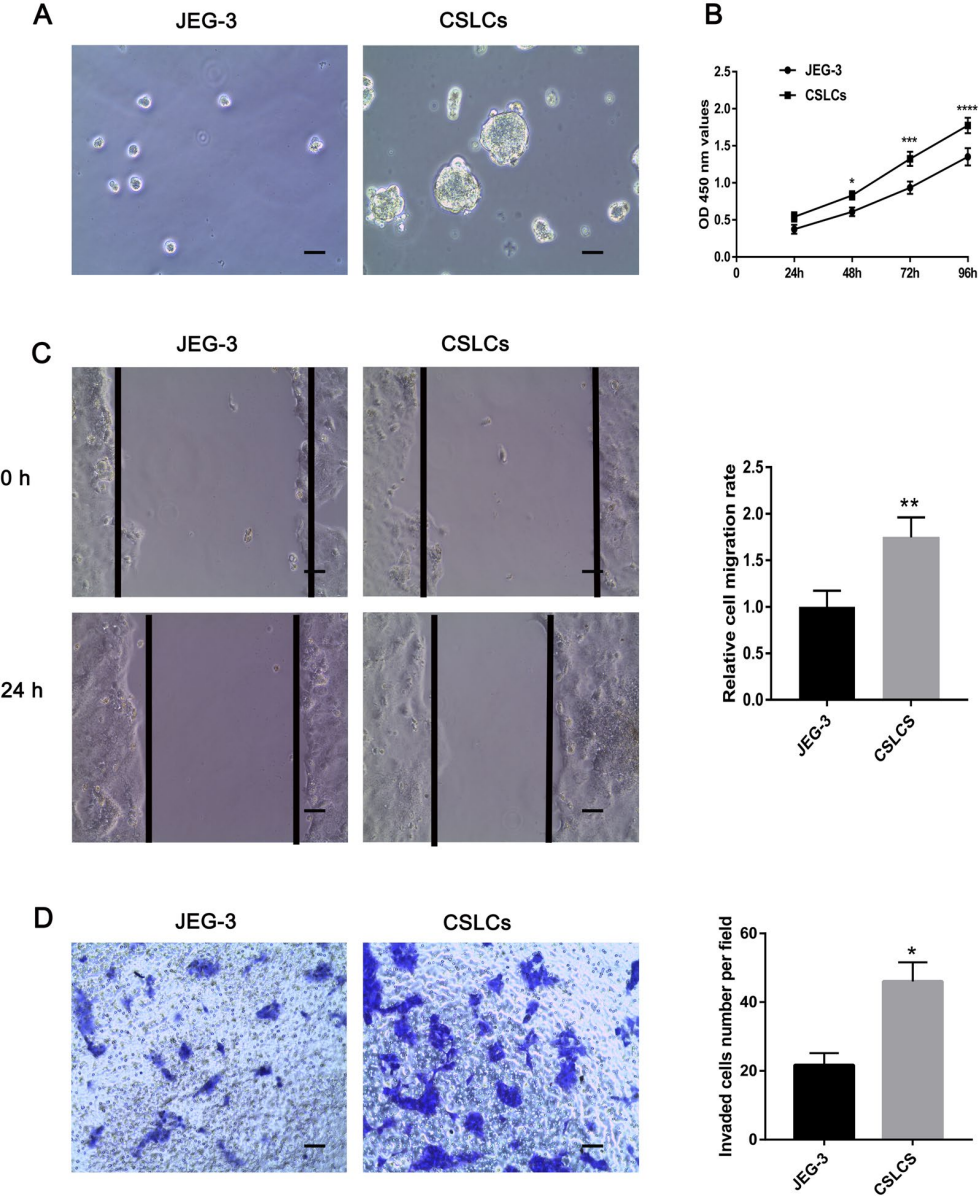
Proliferation, migration and invasion of JEG-3 cells and CSLCs. **A** JEG-3 single-cell suspension and CSLCs cultured in serum-free suspension for 5 days. **B** Proliferation of JEG-3 cells and CSLCs was quantified by using Cell Counting Kit-8 assays. **C** Wound healing assay was performed to evaluate the migration of JEG-3 cells and CSLCs. **D** A Transwell invasion assay was performed using JEG-3 cells and CSLCs. The data are presented as the mean ± SD of three independent experiments. Scale bars = 100 μm. *P < 0.05, **P < 0.01, ***P < 0.001, ****P < 0.001. *CSLC* choriocarcinoma stem-like cells

The current study cultured and identified CSLCs by using the serum-free suspension method [23]. Figure 1A shows the JEG-3 single-cell suspension and CSLCs cultured in serum-free suspension for 5 days. The CCK-8 assay results indicated that the proliferation ability of CSLCs was higher compared with that of JEG-3 cells (Fig. 1B). The wound healing assay results demonstrated that the JEG-3 cell migration rate was significantly lower compared with that of CSLCs (Fig. 1C). For the Transwell invasion assay, the invaded cells were counted in five randomly selected microscopic fields of each experiment and pooled. The invasion of JEG‐3 cells was significantly decreased compared with that of CSLCs (Fig. 1D). These results suggested that CSLCs have a stronger ability of proliferation, migration, and invasion.

### VEGF-B is highly expressed in the CSLCs

**Fig. 2:**
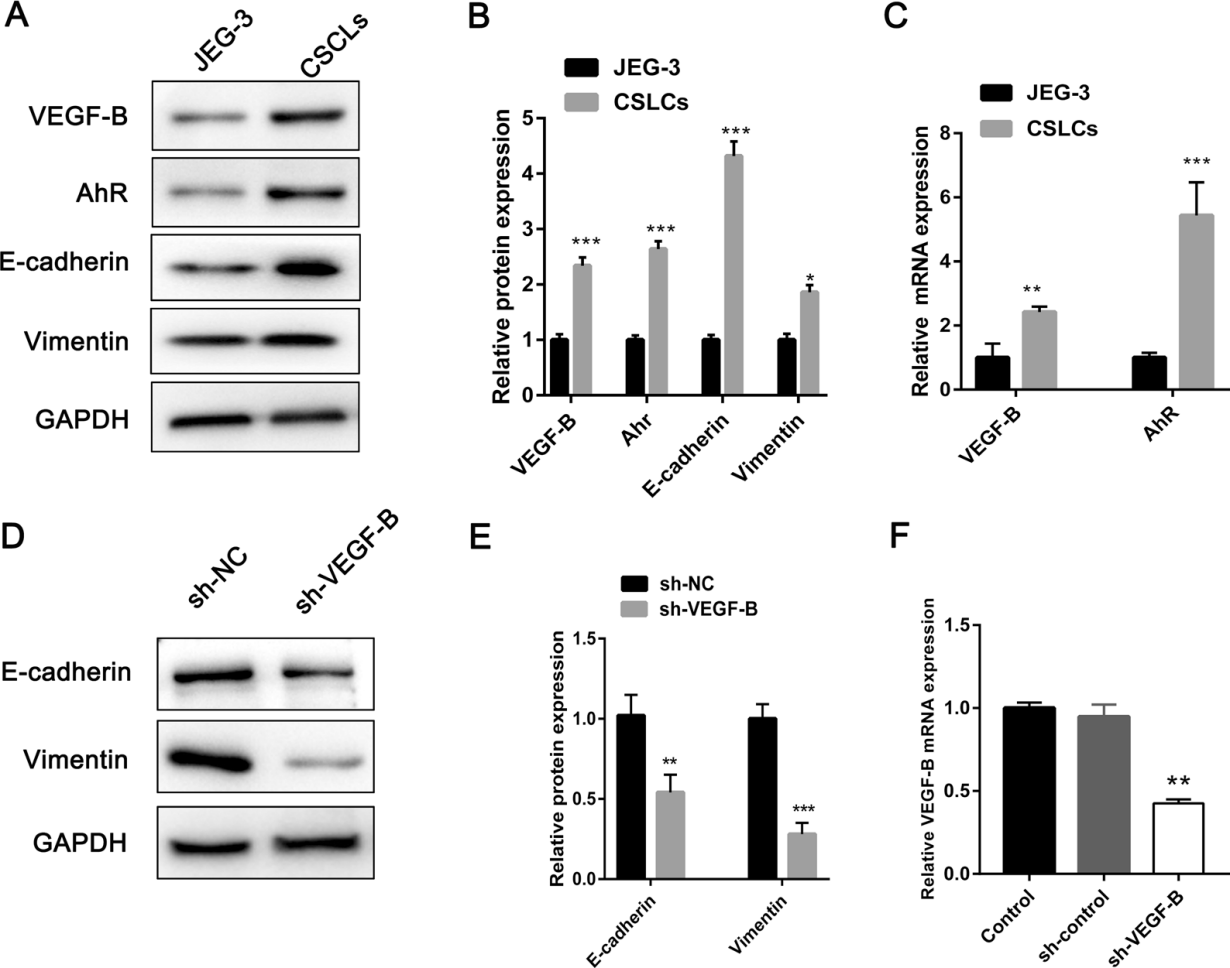
VEGF-B and AhR expression and sh-VEGF-B transfection efficiency. **A** and **B** VEGF-B, AhR, E-cadherin and vimentin protein expression in JEG-3 cells and CSLCs. **C** VEGF-B and AhR mRNA expression in JEG-3 cells and CSLCs. **D** and **E** Western blotting was performed to analyze the differential protein expression levels of E-cadherin and vimentin after VEGF-B knockdown. **F** VEGF-B expression was significantly downregulated in CSLCs by the transfection of VEGF-B shRNA. The results are presented as the mean ± SD (n = 3). **P < 0.01, ***P < 0.001. *CSLC* choriocarcinoma stem-like cells, *shRNA* short hairpin RNA, *AhR* aryl hydrocarbon receptor

Western blot analysis was performed to evaluate VEGF-B protein expression in JEG-3 cells and CSLCs. The expression of VEGF-B protein was upregulated in CSLCs (Fig. 2A, B). The VEGF-B mRNA expression in CSLCs was also increased, compared with that in JEG-3 cells (Fig. 2C).

### VEGF-B knockdown inhibits choriocarcinoma cell proliferation, migration, and invasion

**Fig. 3:**
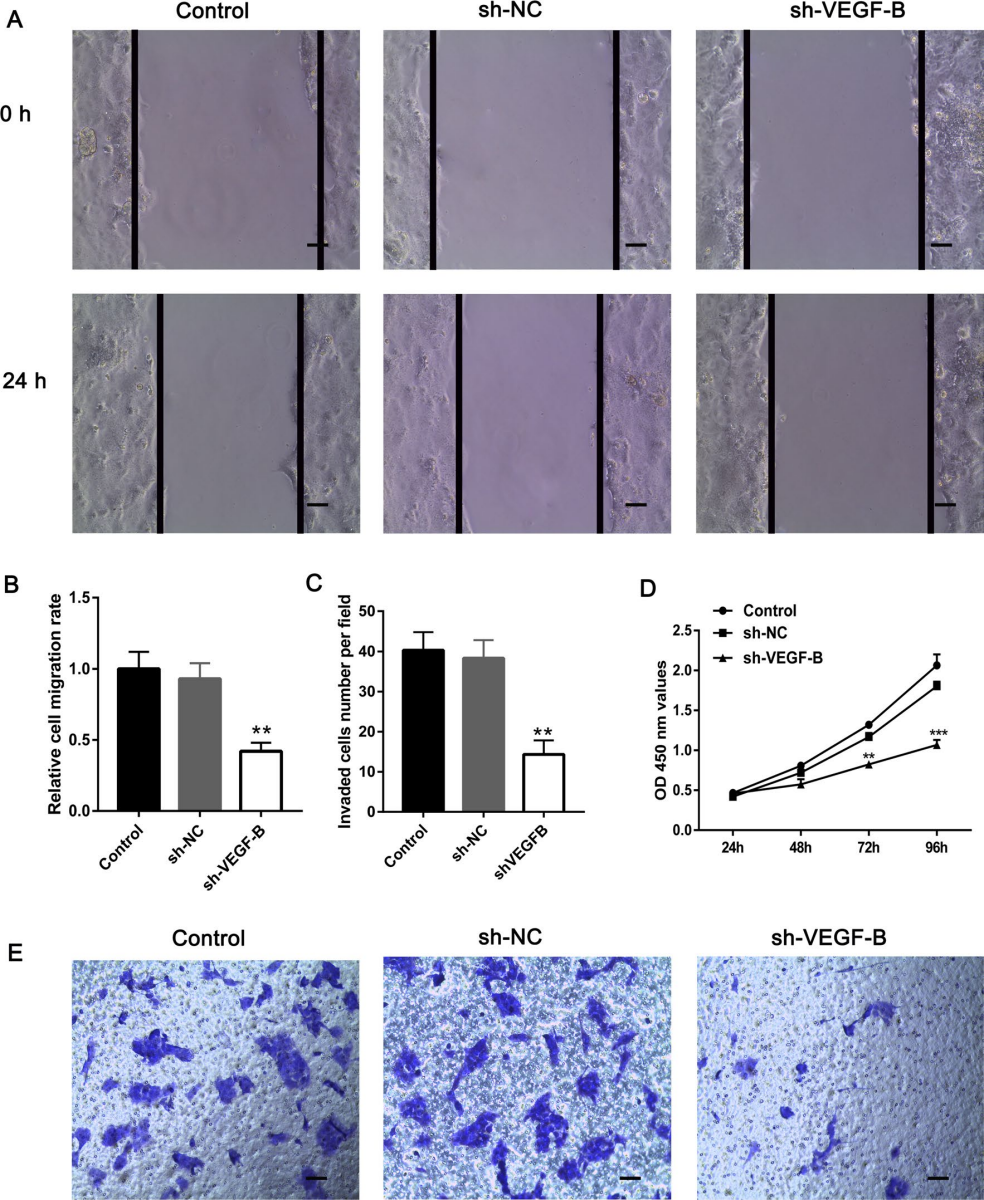
VEGF-B knockdown inhibits CSLC cell proliferation, migration and invasion. **A** and **B** CSLC migration was monitored using wound healing assays in response to sh-VEGF-B-induced VEGF-B knockdown. **D** Cell Counting Kit-8 assay was performed to monitor cell proliferation. **C** and **E** CSLC invasion was monitored using a Transwell invasion assay in response to sh-VEGF-B-induced VEGF-B knockdown. The results are presented as the mean ± SD (n = 3). Scale bars = 100 μm. **P < 0.01, ***P < 0.001. *CSLC* choriocarcinoma stem-like cells, *sh* short hairpin RNA

The expression of VEGF-B was stably knocked down in CSLCs by using VEGF-B shRNA. The differential E-cadherin and vimentin protein expression levels upon VEGF-B knockdown were examined using western blot analysis; the results of which suggesting that E-cadherin and vimentin protein expression was downregulated (Fig. 2D, E). The RT‐qPCR results indicated that the mRNA expression levels of VEGF-B were markedly reduced after transfection (Fig. 2F). A wound healing assay was used to evaluate the migration of CSLCs. After 24 h, the migration rate of the sh-VEGF-B group was significantly decreased (Fig. 3A and B). CCK-8 assays were used to evaluate cell proliferation at 24, 48, 72, and 96 h after seeding. As shown in Fig. 3D, the proliferation of CSLCs was significantly decreased in the sh‐VEGF-B group at 72 and 96 h compared with the sh‐NC group. Moreover, VEGF-B knockdown significantly reduced the invasion of CSLCs (Fig. 3C and E). These results indicated that knocking down VEGF-B inhibited the proliferation, migration, and invasion of CSLCs.

### AhR regulates VEGF-B mRNA and protein expression

**Fig. 4:**
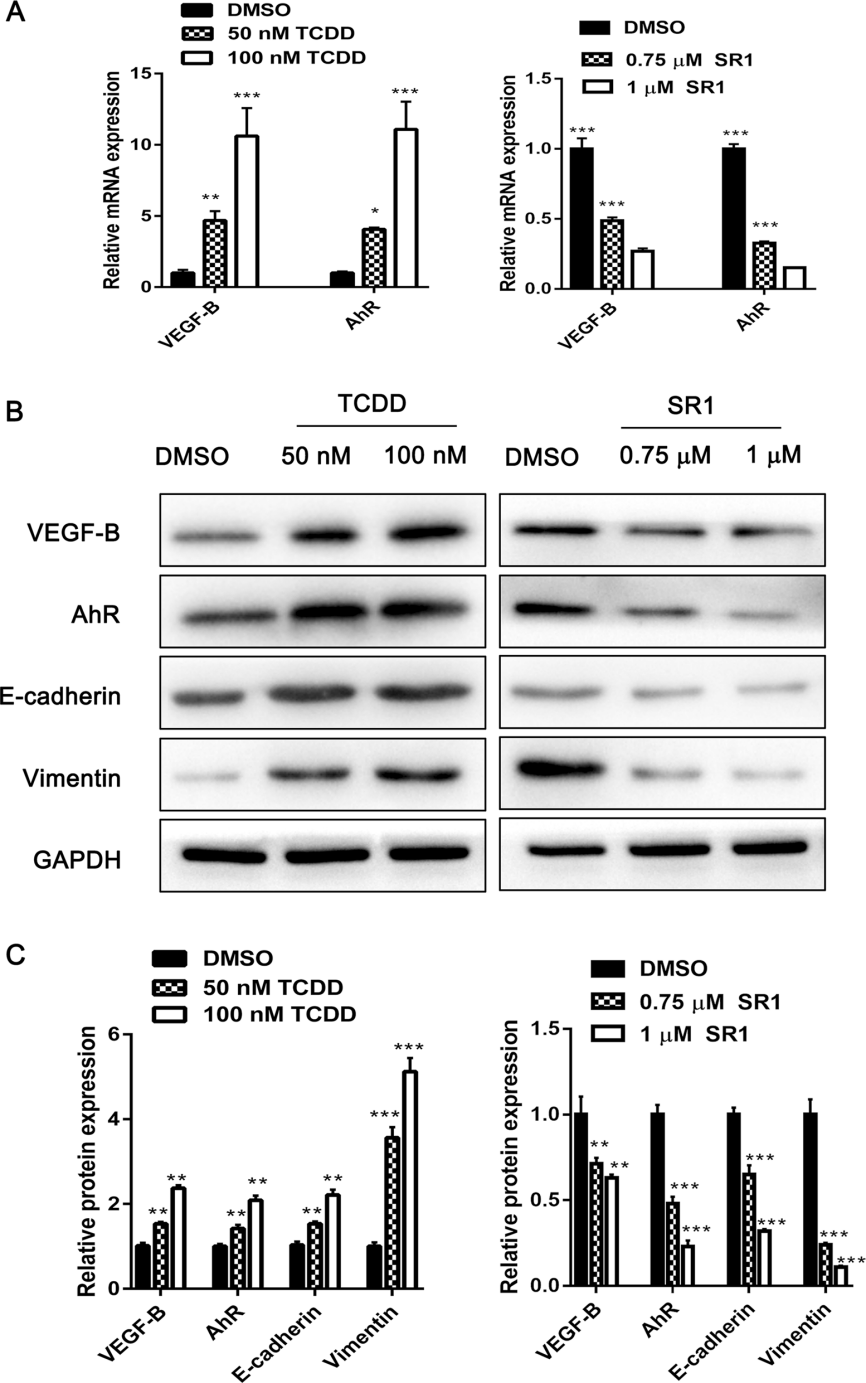
VEGF-B and AhR expression after treatment with TCDD or SR1. **A** CSLCs were treated with or without TCDD and SR1 for 24 h and VEGF-B and AhR mRNA expression was analyzed via reverse transcription-quantitative PCR. **B** Protein expression levels of VEGF-B, AhR, E-cadherin and vimentin were analyzed via western blotting. The protein expression was normalized to GAPDH. **C** Fold change of VEGF-B, AhR, E-cadherin and vimentin protein expression. The results are presented as the mean ± SD (n = 3). *P < 0.05, **P < 0.01, ***P < 0.001. *AhR* aryl hydrocarbon receptor, *TCDD* 2,3,7,8-tetrachlorodibenzo-*p*-dioxin, *SR1* StemRegenin 1, *CSLC* choriocarcinoma stem-like cells

It was found that AhR mRNA and protein were highly expressed in CSLCs (Fig. 2A and B). AhR may be involved in the migration and invasion of choriocarcinoma cells. To investigate the mechanism via which VEGF-B regulates cell migration and invasion, the UCSC genome browser was used and it was found that there were AhR-binding sites in its promoter region. Further experiments were conducted to determine whether AhR could regulate VEGF-B expression. TCDD and SR1 are AhR agonists and antagonists, respectively. As exhibited by the RT-qPCR results, VEGF-B and AhR mRNA expression were significantly upregulated after cells were treated with TCDD in a dose-dependent manner (Fig. 4A, left). However, VEGF-B and AhR mRNA expression was significantly downregulated after cells were treated with SR1 in a dose-dependent manner (Fig. 4A, right). Simultaneously, the western blotting results indicated that the VEGF-B and AhR protein levels were notably upregulated by TCDD treatment, but there was no significant dose-dependent effect (Fig. 4B, C).

### Effects of AhR activation and inhibition on CSLC migration and invasion

**Fig. 5:**
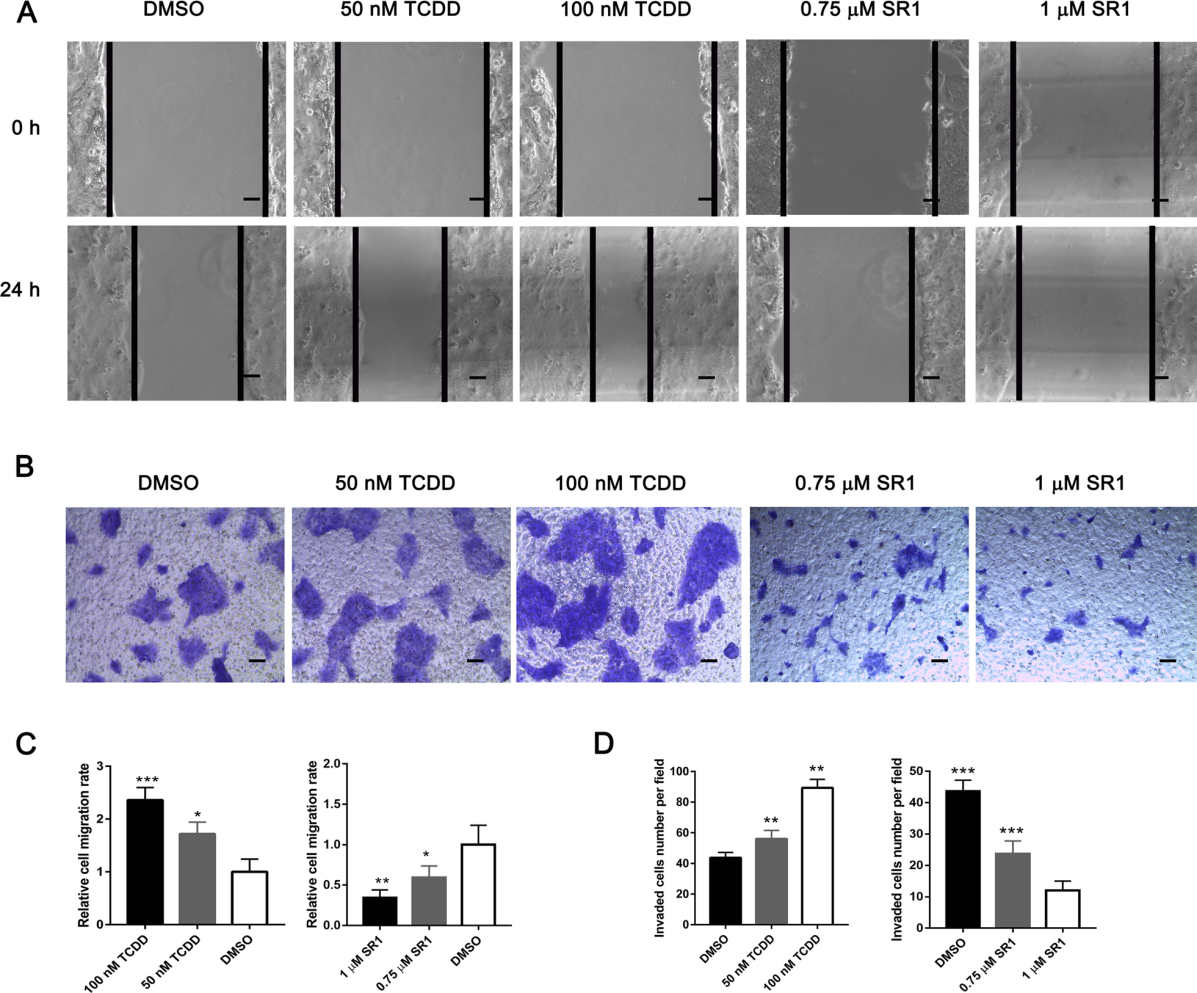
Effects of TCDD and SR1 on the migration and invasion of CSLCs. **A** Wound healing assay was performed to evaluate the migratory properties of CSLCs at two time points (0 and 24 h). **B** Transwell invasion assay was used to analyze the invasive properties of CSLCs at 24 h after cell seeding. The relative **C** migration rate and **D** invading cell number after treatment of CSLCs with TCDD or SR1. The results are presented as the mean ± SD (n = 3). Scale bars = 100 μm. *P < 0.05, **P < 0.01, ***P < 0.001. *AhR* aryl hydrocarbon receptor, *TCDD* 2,3,7,8-tetrachlorodibenzo-*p*-dioxin, *SR1* StemRegenin 1, *CSLC* choriocarcinoma stem-like cells

Wound healing and Transwell invasion assays were performed to examine the effects of AhR activation and inhibition on CSLC migration and invasion. In the wound healing assay (Fig. 5A and C), it was found that cells in the TCDD-treated groups migrated significantly faster compared with the control group, while those in the SR1-treated groups migrated significantly more slowly than the control group. In the Transwell invasion assay (Fig. 5B and D), the invasion of CSLCs of the TCDD-treated groups was significantly increased relative to that of the DMSO-treated control group. However, the invasion of CSLCs of the SR1-treated groups was significantly reduced compared with that of the DMSO-treated control group.

### AhR regulates VEGF-B by direct binding

**Fig. 6:**
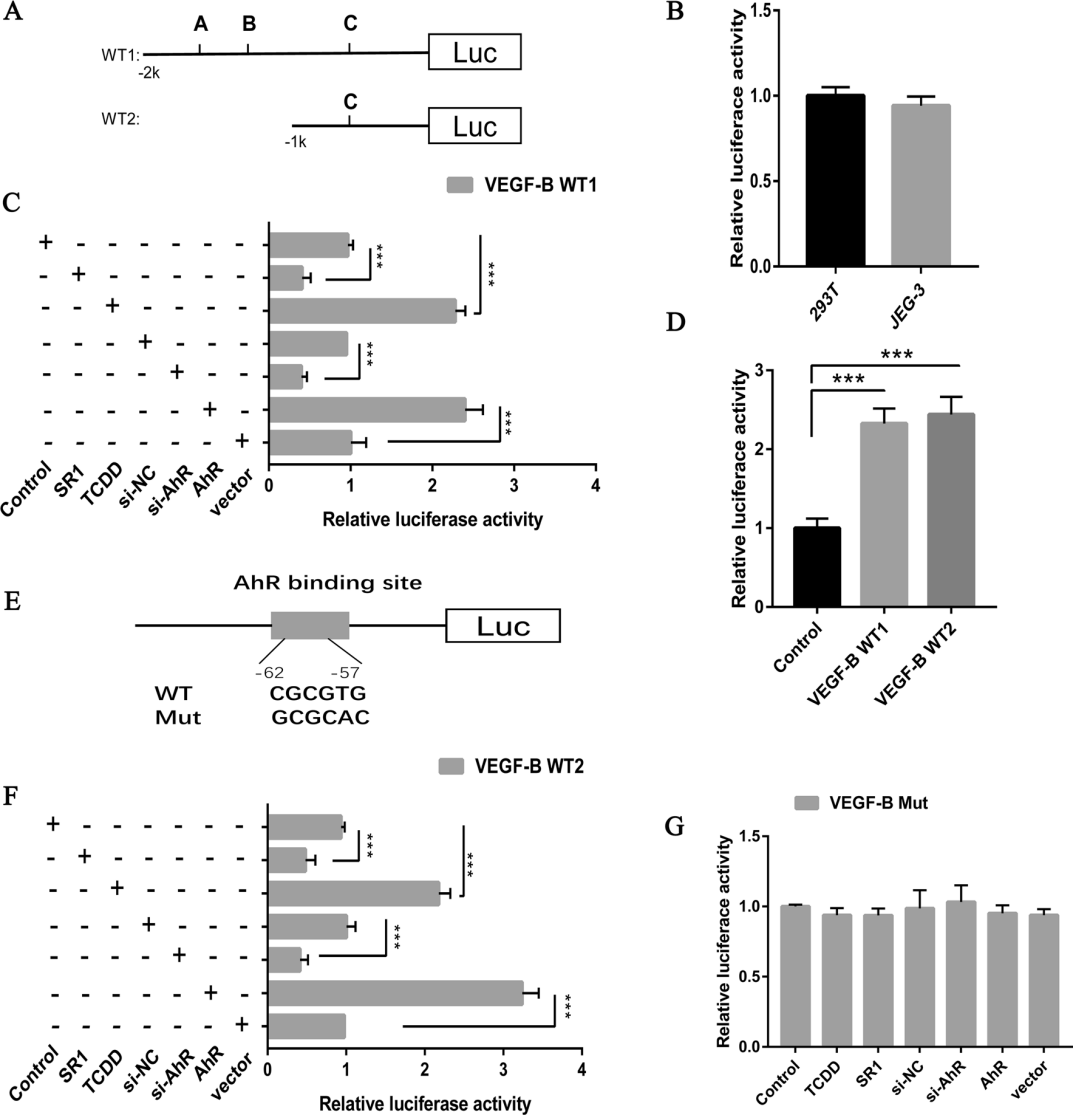
AhR regulates VEGF-B by direct binding. **A** A VEGF-B WT1 (− 2000 to  + 50) luciferase reporter vector and a VEGF-B WT2 (− 1000 to  + 50) luciferase reporter vector were constructed. **B** Relative luciferase activity of 293T and JEG-3 cells. **C** VEGF-B WT1 reporter vector co-transfected with AhR or siAhR, or treated with TCDD or SR1. **D** Luciferase assays were performed using VEGF-B WT1 reporter vector, VEGF-B WT2 reporter vector and negative control. **E** Details of AhR-binding site in VEGF-B promoter. **F** VEGF-B WT2 reporter vector co-transfected with AhR or siAhR, or treated with TCDD or SR1. **G** Luciferase assays were performed using a mutant VEGF-B promoter. The results are presented as the mean ± SD (n = 3). ***P < 0.001. *AhR* aryl hydrocarbon receptor, *TCDD* 2,3,7,8-tetrachlorodibenzo-*p*-dioxin, *SR1* StemRegenin 1, *WT* wild-type, *si* small interfering RNA, *Luc* luciferase

Luciferase reporter gene assays were employed to elucidate the association between AhR and VEGF-B. WT VEGF-B luciferase reporter vectors (VEGF-B WT1 and VEGF-B WT2) were constructed (Fig. 6A). No significant difference in luciferase activity was observed between 293 T and JEG-3 (Fig. 6B). It was demonstrated that TCDD and AhR overexpression increased luciferase activity in JEG-3 cells transfected with VEGF-B WT1. However, SR1 and si-AHR decreased the luciferase activity of JEG-3 cells transfected with VEGF-B WT1 (Fig. 6C). These observations indicate that AhR can bind to the promoter region of VEGF-B. To determine the binding site, another WT luciferase reporter vector, VEGF-B WT2, was constructed. The luciferase activity of VEGF-B WT2 was higher compared with the NC group, while there was no significant difference compared with VEGF-B WT1 (Fig. 6D). The results identified that the binding site was located in VEGF-B WT2. A Mut-type VEGF-B luciferase reporter vector (named VEGF-B Mut) containing a 6-bp mutation in the predicted AhR-binding site was constructed (Fig. 6E). Following co-transfection, the luciferase activity of VEGF-B WT2 was significantly suppressed by si-AhR and SR1, whereas it was amplified by AhR overexpression and TCDD (Fig. 6F). However, after mutation in the predicted AhR-binding site, the luciferase activity changes were abolished (Fig. 6G). Taken together, it was shown that the AhR-binding site was located -62/-57 upstream of the VEGF-B promoter.

**Fig. 7:**
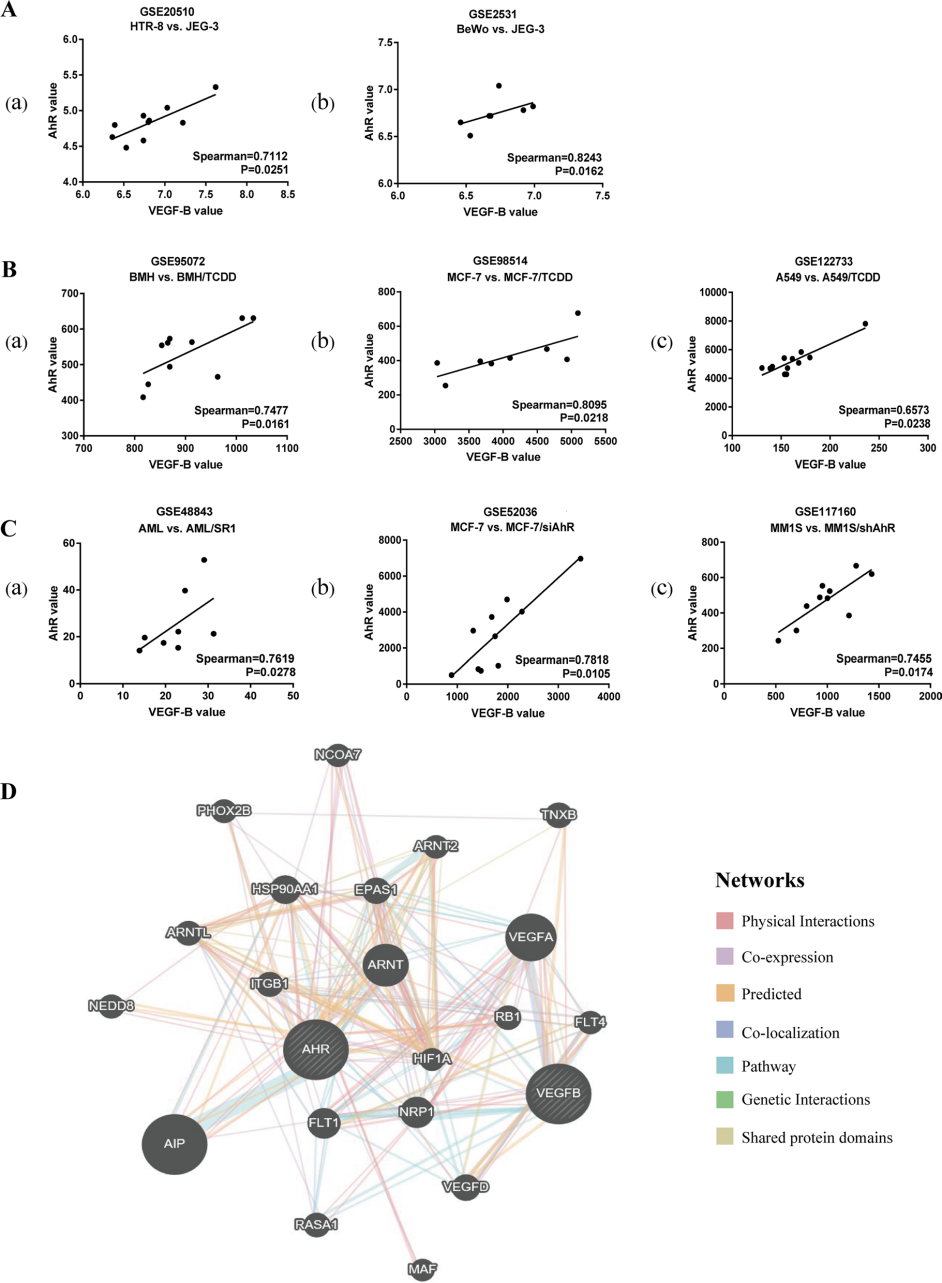
AhR expression positively correlates with the levels of VEGF-B. **A** AhR and VEGF-B mRNA expression levels in trophoblast cell lines. (a) Choriocarcinoma JEG-3 cells vs. extravillous trophoblast HTR-8 cells in GSE20510; (b) choriocarcinoma JEG-3 cells vs. BoWo cells in GSE2531; **B** AhR and VEGF-B mRNA expression levels in AhR agonist treated cells vs. parent cells. (a) For mesenchymal stem cells, BMH cells, in GSE95072. (b) For breast cancer MCF-7 cells in GSE98514; (c) for lung cancer A549 cells in GSE122733. **C** AhR and VEGF-B mRNA expression levels in AhR antagonist or AHR knockdown cells vs. parent cells. (a) For primary human acute myeloid leukemia cells in GSE48843; (b) for breast cancer MCF-7 cells in GSE52036; (c) for multiple myeloma MM1S cells in GSE117160. **D** Molecular functional network map of AhR and VEGF-B was analyzed using the GeneMANIA tool. *AhR* aryl hydrocarbon receptor

Due to the limited data regarding choriocarcinoma, the GEO datasets were used to conduct bioinformatics analysis on different cell lines intervened by AhR agonists, AhR antagonists, or AhR knockout. In choriocarcinoma cell lines, it was found that the expression levels of VEGF-B and AhR were positively correlated (Fig. 7A). Furthermore, the results demonstrated that the expression patterns of VEGF-B and AhR in different cell lines were similar, whether with AhR activation or inhibition (Fig. 7B, C). The online GeneMANIA tool was used to observe the possible molecular pathway of AhR and VEGF-B. The networks identified by GeneMANIA are presented in Fig. 7D. As shown in Table 2, five genes [aryl hydrocarbon receptor interacting protein, integrin subunit β 1, NRP1, hypoxia inducible factor 1 subunit α (HIF1A), and endothelial PAS domain protein 1] were found to be directly associated with both VEGF-B and AhR. Collectively, these results indicated the co-expression correlation of VEGF-B and AhR.

**Table 2:** Possible genes related to VEGF-B/AhR signaling axis

Gene	Full name
AIP	Aryl hydrocarbon receptor interacting protein
ITGB1	Integrin subunit beta1
NRP1	Neuropilin 1
HIF1A	Hypoxia inducible factor 1 alpha subunit
EPAS1	Endothelial PAS domain protein 1

## Discussion

Although most patients with choriocarcinoma are cured by chemotherapy under the guidance of the sensitivity response marker human chorionic gonadotropin [4], several studies have reported the mortality rate of patients with brain metastasis of choriocarcinoma after treatment is 15–50% [30–32]. Therefore, exploring the migratory and invasive mechanisms of choriocarcinoma has an important clinical significance for the development of targeted treatment and improvement of the prognosis of patients with metastasis.

CSCs and their interaction with the tumor microenvironment are critical throughout metastatic progression [33]. Evidence suggests that CSCs are present in the blood of patients with breast cancer; when inoculated into immune-deficient mice, these cells can produce bone, liver, and lung metastases [34]. A mouse model of colorectal cancer showed that selective Lgr5^+^ CSC ablation inhibits primary tumor growth and distinct CSC dependencies for primary vs. metastatic tumor growth [35]. Moreover, it has been suggested that the VEGF autocrine signaling pathway is mediated by the receptor tyrosine kinase VEGFR2 and NRPs can regulate the self-renewal ability and the number of CSCs. The VEGF autocrine pathway is active in tumor metastasis and poorly differentiated tumors [12, 36, 37]. CSCs have been hypothesized to represent the driving force underlying tumor progression and metastasis, making them attractive treatment targets. However, conclusive experimental evidence for their functional relevance is lacking for choriocarcinoma. In the present study, CSLCs were shown to have higher proliferation, invasion, and migration abilities compared with JEG-3 cells. The results indicated that CSLCs may be involved in the growth and metastasis of choriocarcinoma.

VEGF-B has been reported to be minimally angiogenic among VEGF family members. A peptide designing strategy based on the receptor-binding segments of VEGF-B effectively inhibits angiogenesis, tumor growth, and metastasis in BALB/c mice [38]. Moreover, the elevation of VEGF-B is correlated with metastasis in a nude mouse model of colorectal cancer [19]. Our previous study revealed that JEG-3 cells highly expressed VEGF-B and had a higher invasion ability compared with JAR cells [39]. The detailed function of VEGF-B in choriocarcinoma remains to be discovered. The present study first observed a notably higher VEGF-B expression in CSLCs compared with JEG-3 cells, suggesting that there may also be post-translational modifications to VEGF-B. Following the knockdown of VEGF-B by shRNA transfection, CSLC proliferation was markedly hindered. In addition to tumor cell proliferation, the migratory and invasive abilities of cancer cells were significantly suppressed by VEGF-B knockdown, indicating the potential role of VEGF-B in choriocarcinoma cell migration and invasion in vitro. Based on these results, it could be suggested that VEGF-B may function as an oncogene in choriocarcinoma, consistent with the aforementioned study.

In the present study, VEGF-B knockdown also suppresses E-cadherin and vimentin expression. Epithelial-mesenchymal transition (EMT) is usually accompanied by a decrease of E-cadherin levels [40]. However, Hollestelle et al. [41] reveal that, in human breast cancer, loss of E-cadherin expression is not causal or necessary for EMT. Numerous metastatic tumors still contain high levels of E-cadherin and epithelial cancer cells expressing E-cadherin can invade and metastasize without undergoing complete EMT [42–44]. These are consistent with the present results. Subsequent assays were conducted to identify the mechanism underlying the impact of VEGF-B on choriocarcinoma cell migration and invasion.

Increasing evidence has shown that AhR is involved in regulating cell adhesion and their migration potential [21]. Previous studies have identified that increased AhR activity augments cell migration in lung cancer and gastric cancer via a mechanism involving JNK activation [45, 46]. A previous study also reported a role for the AhR/cytochrome P450 1A1 pathway in breast CSC expansion [47]. By contrast, the expression of the chemokine C-X-C motif chemokine receptor 4 is downregulated by AhR agonist-mediated activity in breast cancer cells, suggesting an anti-metastatic effect of AhR activation [48]. However, the exact molecular mechanisms remain to be elucidated. The present study found a significant upregulation of AhR and VEGF-B expression by the AhR agonist TCDD in CSLCs. By contrast, SR1 downregulated AhR and VEGF-B expression in CSLCs. Moreover, SR1 markedly suppressed choriocarcinoma cell proliferation, migration, and invasion, while TCDD exerted the opposite effect. These results indicated that AhR may serve as an oncogene in choriocarcinoma and it may regulate CSLC proliferation, migration, and invasion by targeting VEGF-B. To the best of the authors’ knowledge, the present was the first study to evaluate the correlation between VEGF-B and AhR. Notably, using the UCSC database, it was found that AhR possibly binds to the VEGF-B promoter. Furthermore, luciferase reporter vector assays were performed to confirm that AhR regulated VEGF-B expression via direct targeting. All these results suggested that VEGF-B mediated choriocarcinoma cell migration and invasion by targeting AhR. It has previously been reported that TCDD acts directly on C57BL/6 mouse ocular tissues via AhR to promote VEGF-B mRNA expression [49], which is consistent with the current results.

There is currently a lack of big data for choriocarcinoma owing to the difficulty in obtaining tissue samples. The present study analyzed the correlation of VEGF-B and AhR in different cells, including breast cancer, primary human acute myeloid leukemia, and lung cancer by using GEO data. Despite the small sample sizes, the results demonstrated a positive correlation between VEGF-B and AhR, further verifying the current findings. In the predicted molecular pathways, the current study found some interesting factors directly associated with both VEGF-B and AhR, such as HIF1A and NRP1, which indicated that additional research should be performed to investigate the regulatory network of the VEGF-B/AhR axis. VEGF/NRP signaling may serve a role in the function of CSCs and tumor development [13]. Genetic deletion of NRP1 can prevent the ability of VEGF to promote cell stemness and self-renewal [50]. A previous study demonstrates the importance of VEGF/VEGFR2/NRP1 signaling in the viability, self-renewal, and tumorigenicity of glioma CSCs [51]. These mechanisms may explain the current experimental results.

In summary, to the best of the authors’ knowledge, the present study was the first to provide evidence that VEGF-B could promote choriocarcinoma cell migration and invasion by direct AhR targeting. It is probable for the VEGF-B/AhR axis mentioned in the present study to be functional in preventing the migration and invasion of choriocarcinoma cells, which may be a possible therapeutic strategy for choriocarcinoma.

## Supplementary Information

**Additional file 1: Table S1**. The sequence of VEGF-B short hairpin RNA and AhR small interfering RNA.

## Data Availability

The datasets used and/or analyzed during the present study are available from the corresponding author on reasonable request.
