# Supplementary material for: VEGF-B targeting by aryl hydrocarbon receptor mediates the migration and invasion of choriocarcinoma stem-like cells
Source: Cancer Cell Int. 2022 Jun 30;22:221. doi: 10.1186/s12935-022-02641-8 (PMC9245252; doi:10.1186/s12935-022-02641-8)
Supplement: Supplementary file 1 — Additional file 1: Table S1. The sequence of VEGF-B short hairpin RNA and AhR small interfering RNA. [file 12935_2022_2641_MOESM1_ESM.docx]

Supplemental table I. The sequence of VEGF-B short hairpin RNA and AhR small interfering RNA.

|  | Sequence (5′- 3′) |
| --- | --- |
| sh-VEGF-B | GATCCACAGTCAGCCATAATA |
| si-AhR | GCAACAAGATGAGTCTATTTA |
